# Investigation on an outbreak of cutaneous anthrax in a county of Shandong Province, China, 2021

**DOI:** 10.1186/s12879-022-07802-8

**Published:** 2022-11-22

**Authors:** Xiaolin Yu, Ming Fang, Shuang Wang, Zhong Li, Lixiao Cheng, Zhaoshan Liu, Dandan Zhang, Dandan Dong, Zengqiang Kou

**Affiliations:** 1 Shandong Provincial Center for Disease Control and Prevention, Jinan, Shandong China; 2 Chinese Field Epidemiology Training Program, Chinese Center for Disease Control and Prevention, Beijing, China; 3 Cao County Center for Disease Control and Prevention, Heze, Shandong China; 4 Heze Center for Disease Control and Prevention, Heze, Shandong China

**Keywords:** Cutaneous anthrax, Outbreak

## Abstract

**Background:**

In order to verify the existence of an anthrax outbreak, determine its scope, grasp the epidemiological characteristics and find out the cause of the outbreak and recommend preventive and control measures.

**Methods:**

Etiological hypothesis was developed through descriptive epidemiological methods. Hypotheses were tested by analyzing epidemiological methods by comparing the differences in the incidence of different exposure types. Nucleic acid detection and bacterial isolation and culture in the BSL-2 laboratories. SPSS 21 was used to conduct statistical analysis.

**Results:**

A total of 126 family, workshop, shop environment samples and meat samples were collected, and 6 samples were collected from skin lesions of suspected cutaneous anthrax cases. 41 samples were positive by rPCR and 8 strains of Bacillus anthracis were cultivated. Participated in slaughtering, cutting beef of sick cattles was significantly associated with cutaneous anthrax (RR 3.75, 95% CI 1.08–13.07), this behavior is extremely dangerous.

**Conclusions:**

Comprehensive analysis of laboratory results and epidemiological survey results and environmental assessments, we judge this epidemic to be an outbreak of cutaneous anthrax, associated with slaughtering and other processes from infected cattle imported from other province.

## Introduction

Anthrax is an acute zoonotic disease caused by the gram-positive spore-forming bacteria Bacillus anthracis [1]. Bacillus anthracis is environmentally stable in spore form and may contaminate soil, resulting in infections of herbivores while grazing [1]. Human transmission occurs via contact with infected animals through butchering and working with hides or ingestion of raw or undercooked meat [2]. Humans may develop cutaneous, inhalational and gastrointestinal infection. Cutaneous anthrax accounts for 95% of human cases and is characterized by itching and skin lesions starting 1–7 days after infection; the lesions eventually form depressed eschars [1]. Anthrax is acquired from animals; there are rare reports of direct human to human transmission [3].

Anthrax occurs worldwide, and the World Health Organization (WHO) estimates the annual global incidence of between 2000 and 20,000 cases [4]. Study shows that during 1955–2014, a total of 120,111 probable and confirmed human anthrax cases, including 4341 fatal cases, were reported to the China CDC; the overall case-fatality rate was 3.6%. Before the 1980s, probable and confirmed human anthrax incidence showed a periodic increase and decrease every 8–10 years. Thereafter, incidence decreased until 2013, when it reached a low of 193 cases (0.014 cases/100,000 population) [5]. Since 1996, no more than 10 cases have been reported every year in Shandong province, no cases were reported from 2015 to 2020. No cases have been reported since 1995 in the county where the outbreak occurred.

On August 11 and 18, 2021, the Public Health Clinical Center reported to the CDC a total of 7 clinically diagnosed cases of cutaneous anthrax from one county, including 3 and 4, respectively. The investigation team went to the C county where the case was located to conduct an investigation immediately, to verify the existence of an anthrax outbreak, determine its scope, grasp the epidemiological characteristics and find the cause of the outbreak, recommend preventive and control measures.

## Methods

### Investigation area

**Fig. 1:**
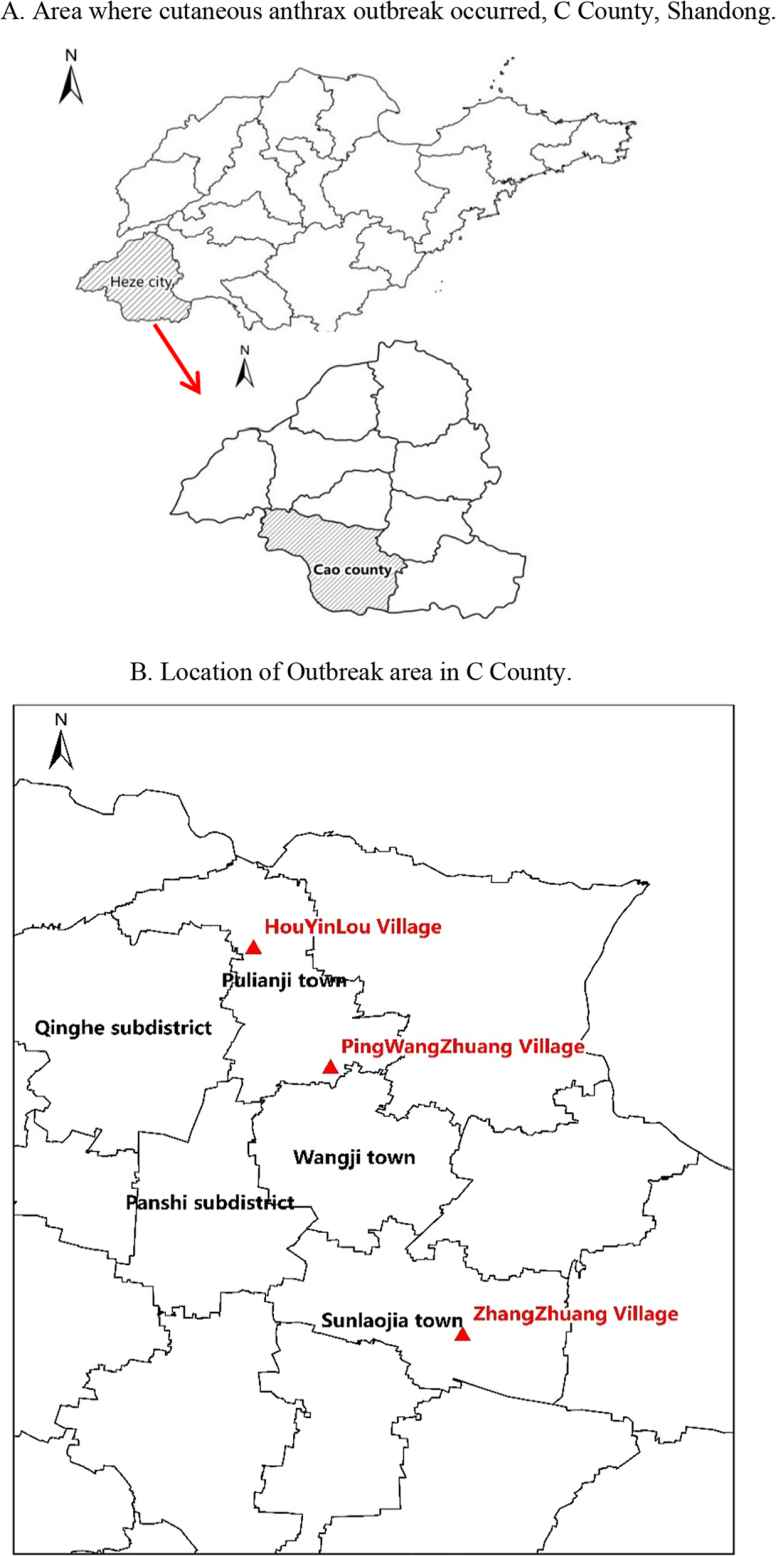
**A** Area where cutaneous anthrax outbreak occurred, C County, Shandong. **B** Location of Outbreak area in C County

The outbreak cases all occurred in one county-Cao County (C County), it’s in the southwest of Heze City. Location of the outbreak in the northeast of the county involving two towns and three villages, Houyinlou Village (H Village) and Pingwangzhuang Village (P Village) in Pulianji Town, Zhangzhuang Village (Z Village) in Sunlaojia Town (Fig. 1).

### Case definition

For this investigation, we defined a suspected cutaneous anthrax case as since July 14, 2021, those who have been in contact with sick or dead animals or their products, or have eaten meat or their products from sick or dead animals, or inhaled suspected Bacillus anthracis contaminated dust, or engaged in occupation that is in close contact with animal products such as fur, at the same time, people who have one of the symptoms of unexplained macules, papules, blisters, swelling, ulcers or eschar on the skin of exposed parts such as hands, forearms, face, and neck.

A confirmed case was a suspected case followed up with a clinical specimen (blood or swab from skin lesion or vesicle) that tested positive for Bacillus anthracis by rPCR, also tested positive for serological antibodies. The case definitions were coincident with “Diagnosis for anthrax” (WS 283-2020, China National Health Commission).

### Case search

To search for suspected cases as quickly as possible we check the medical records of medical institutions include county hospitals, township health centers and village clinics. Household surveys and visits were carried out in the villages where the cases occurred to find co-exposed persons and close contacts. Medical institutions and clinics at all levels in C County are required to report suspected cases on a daily basis after August 20, 2021. Six suspected cases were identified through active searches and confirmed by laboratory tests. The outbreak involved a total of 13 cases.

### Ethics statement

The study was approved by the Ethics Committee of Shandong CDC, all patients provided written informed consent.

### Descriptive epidemiology and hypothesis generation

The etiological hypothesis was formed by analyzing the onset time, location and demographic characteristics of the cases, Possible exposure factors were identified by interviewing patients and close contacts. Through epidemiological investigation, it was found that all the cases were engaged in cattle-related work such as slaughtering, cutting beef, buying beef and recycling tallow. They are all related to the cattle or beef of Wang’s family in Z Village, S Town.

### Hypothesis verification

By comparing the differences in the attack rate of different types of exposure to analyze the differences in the risk rates of different behaviors, we evaluated the association between exposure to the suspicious cow and illness onset, calculate the incidence rate. We computed the risk ratio (RR) for each activity that resulted in exposure to assess the association between each individual exposure and subsequent illness.

### Laboratory investigations

Dip the blister fluid with two sterile cotton swabs or apply it repeatedly on the skin lesions and skin eschar, and immediately send it to the laboratory for testing under the condition of 2–8 °C storage. The collected specimens should be placed in sealable sampling tubes and other containers, and double-packed to avoid spillage during transportation.

According to the National Anthrax Surveillance Program (Trial), the reported cases of anthrax were investigated, and samples of patients, suspicious livestock materials (organs, blood and fur of diseased or dead animals), and samples of contaminated environment (such as soil and water) were collected for testing. A total of 126 family, workshop, shop environment samples and meat samples were collected, and 6 samples were collected from skin lesions of suspected cutaneous anthrax cases. Nucleic acid detection and bacterial isolation and culture in the BSL-2 laboratories. According to Diagnosis for anthrax (WS 283-2020, China National Health Commission) PCR test conform to the laboratory tests for confirmed cases. The kit was purchased from Shanghai Biogerm Biotechnology Company. The confirmed cases of cutaneous anthrax in this investigation were confirmed by PCR test combined with serum antibody testing.

## Results

### Descriptive epidemiology and hypothesis generation

We identified 13 cases of cutaneous anthrax, 7 clinically diagnosed cases and 6 confirmed by rPCR testing, there were no severe cases or deaths. The cases were all adults and the mean age was 50 (range 28–68) years, the male to female ratio was 12:1. The main clinical manifestations were anthrax carbuncle and fever, anthrax carbuncle is most manifested in the form of skin reddening, erythema, papule and vesicles, edema. Anthrax carbuncle location is mostly in the upper limb, the number of anthrax carbuncle is mostly one (Table 1).

**Table 1:** Demographic information and clinical characteristics of the cases during an outbreak, C County, Shandong, August 2021

Classification	Number of cases (N = 13)	Percentage (%)
Sex		
Male	12	92.3
Female	1	7.7
Age		
25~	1	7.7
35~	3	23.1
45~	7	53.8
55~	1	7.7
65~	1	7.7
Signs and symptoms		
Skin reddening/erythema/papule	13	100
Skin vesicles/edema	13	100
Skin ulceration/eschar	7	53.8
Skin itching/pruritis	4	30.8
Fever	2	15.4
Skin vesicles/edema and Fever	2	15.4
Cough	0	0
Headache	0	0
Diarrhea	0	0
Anthrax carbuncle site		
Upper limbs	11	84.6
Finger	2	15.4
Back of the hand	1	7.7
Lower limbs	1	7.7
Number of anthrax carbuncle		
4	1	7.7
3	3	23.1
2	2	15.4
1	7	53.8

We analyzed the geographic locations of the cases, 6 (46%) lived in H Village and 3 (23%) lived in P Village, 4 (31%) lived in Z Village. The three villages are not close to each other, According to the investigation, three families in three villages had slaughtered infected cattle. Investigations found that the cases were all related to cattles of Wang’s farm in Z Village. Wang said that he had recently purchased 32 cows from Y Town, J Province, of which 20 cows have been in poor health since July, and the address of the animal husbandry quarantine certificate he provided does not match, so the source of the cattle was privately traded cattle without quarantine certificates. Starting in July, 20 cattles in his farm were found to be in poor health, the cattles were not eating well, and the spirit is not good, and he asked veterinarians from other places to diagnose that the cattles might be sick, so he then dealt with the suspected infected cattle.

**Fig. 2:**
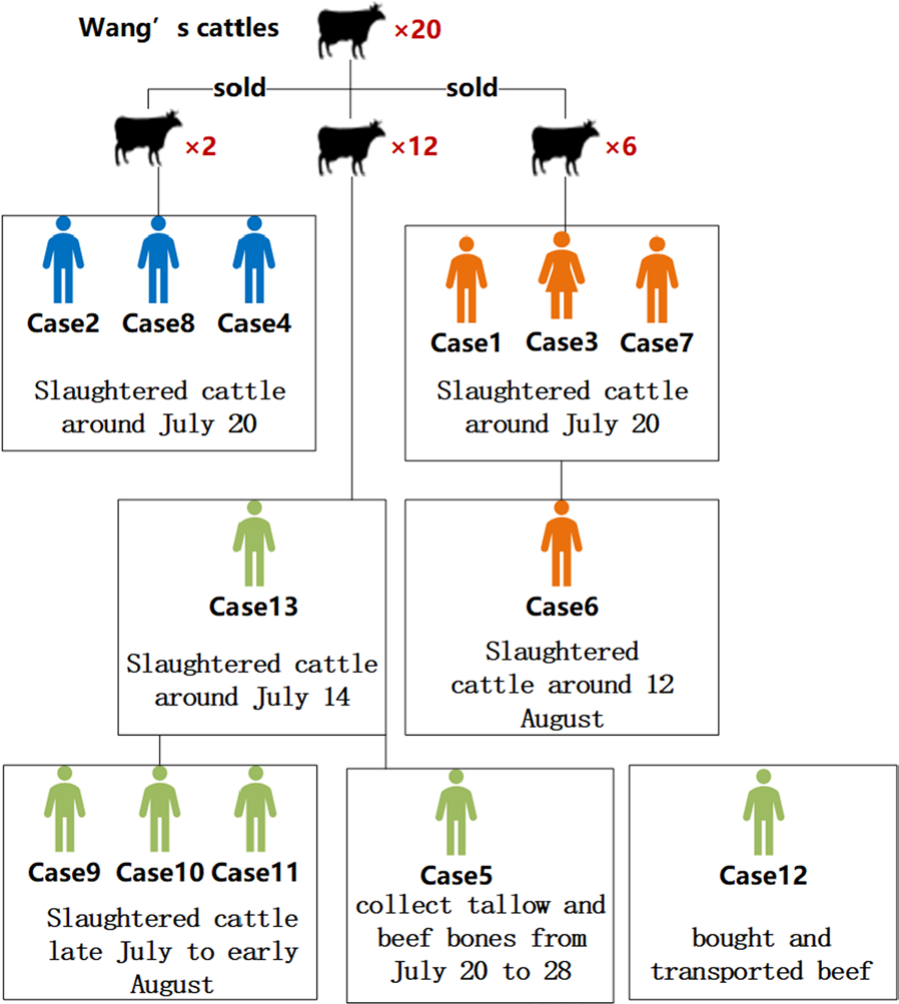
Case diagram during an outbreak that occurred in August 2021, C County, Shandong

Wang successively sold 2 of the 20 cattles to case 8 in P Village, sold 6 of the 20 cattles to case 7 in H Village, and Wang invited others to slaughter remaining 12 cattles. Case 1 and Case 3 went to case 7’s home for slaughter around July 20, Case 7 participated in transporting beef. Case 6 slaughtered cattle from case 7’s family on 12 August. Case 2 and Case 4 went to case 8’s home for slaughter around July 20, Case 8 participates in slaughtering activities. Two of wang’s 12 cattles were slaughtered by case 13 on July 14–15, the remaining were slaughtered from late July to early August by case 9, case 10 and case 11, Case 5 went to Wang’s home to collect tallow and beef bones from July 20 to 28, and participated in handling and processing. Case 12 bought and transported beef from Wang’s farm on August 5. All the cases have been linked to the 20 sick cattles (Fig. 2).

**Fig. 3:**
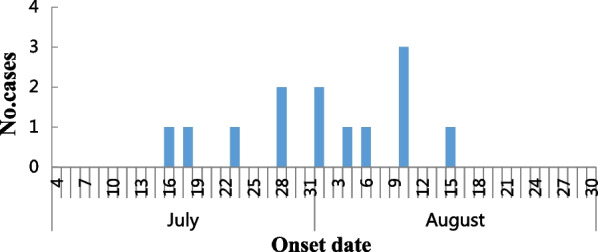
Distribution by date of onset of cases during an outbreak that occurred in August 2021, C County, Shandong

An epidemic curve was drawn based on the onset time of 13 cases, the epidemic curve showed that, the first case occurred on July 16, and the last case occurred on August 15, the median incubation period is 5 days (2–16), showing the characteristics of a continuous exposure epidemic curve (Fig. 3), consistent with the investigation.

### Hypothesis verification

The attack rate of slaughtering, cutting beef was 83.3%, collecting tallow and beef bones was 33.3%, handling and transporting beef, offal, cowhide was 22.2%. In the comparing the differences in the attack rate, certain activities were significantly associated with developing cutaneous anthrax: Use the form with the lowest attack rate as a reference, participated in slaughtering, cutting beef of sick cattles was significantly associated with cutaneous anthrax (RR 3.75, 95% CI 1.08–13.07), this behavior is extremely dangerous (Table 2). And during the process of slaughter or contact with cattle, the participants have no protection, increased risk of infection.

**Table 2:** Prevalence of different exposures during outbreak, C County, Shandong, August 2021

Form	Cases		Attack rate (%)		RR (95%CI)
	Exposed	Nonexposed	Exposed	Nonexposed	
Participated in slaughtering/cutting beef	10	0	83.3	0	3.75 (1.08–13.07)
Participated in collecting tallow and beef bones	1	0	33.3	0	1.50 (0.20–11.24)
Participated in handling and transporting beef/offal/cowhide	2	0	22.2	0	Ref
Ate cooked beef or sweetbreads	0	0	0	0	0
Lived with patients	0	0	0	0	0
Treated cases	0	0	0	0	0

### Laboratory test results

Skin lesion swabs collected from 7 hospitalized patients, 1 tested positive for Bacillus anthracis DNA by rPCR. It should be noted that, at the time of specimen collection, all patients had already started and some had completed antimicrobial treatment. Five skin lesion swabs of 6 cases identified by active search were tested positive. A total of six cases tested positive.

All cases’ homes, cold storages, places where beef and other products were sold, and all equipment, utensils and meat products in the places were sampled. A total of 125 samples of environment and utensils and 1 meat sample were collected during the entire investigation. 34 samples were positive by rPCR and 8 strains of Bacillus anthracis were cultivated. A beef sample from case 8’s cold storage tested positive, and the beef is from Wang’s cattle. 33 environmental and appliance samples were positive, including Wang’s cattle farm sewage, the ground where cattle used to be slaughtered, cattle trucks daub sample, Case 8’s yard ground where cattle used to be slaughtered, sewage, Case 8’s beef store’s cold storage, meat basin, cutting board, meat grinder, weighing scale. Case 7’s yard ground where cattle used to be slaughtered, cow saws, cold storage and butcher shop environment (meat mincer, freezer inner wall daub, bone cutter daub, cutting board, ground, freezer handle), plate of barbecue shops where Case 7’s meat was sold. Case 6’s yard ground, Case 6’s butcher shop floors, door handles, meat hooks, bone saws, and meat carts, Case 11’s cattle slaughter knife. A total of 8 strains of Bacillus anthracis were cultured (Table 3).

**Table 3:** Laboratory test results of environmental samples during an outbreak, C County, Shandong, August 2021

	Sample type	Sample source	PCR results	Bacterial culture results
1–3	Environmental samples	Wang’s cattle farm sewage, the ground where cattle used to be slaughtered, cattle trucks daub sample	**+**	2 strains of bacteria (cattle trucks daub sample)
4–13	Environmental samples	Case 8’s yard ground where cattle used to be slaughtered, sewage, cold storage, etc	**+**	1 strain of bacteria (yard ground)
14	Beef	A beef sample from case 8's cold storage	**+**	/
15–26	Environmental samples	Case 7’syard ground where cattle used to be slaughtered, cow saws, cold storage and butcher shop environment, etc	**+**	2 strains of bacteria(cow saws, Inner side wall of cold storage)
27	Environmental samples	Plate of barbecue shops where Case 7’s meat was sold	**+**	/
28	Environmental samples	Case 6’s yard ground	**+**	1 strain of bacteria
29–33	Environmental samples	Case 6’s butcher shop floors, door handles, meat hooks, bone saws, and meat carts	**+**	/
34	Environmental samples	Case 11’s cattle slaughter knife	**+**	2 strains of bacteria

## Discussion

Comprehensive analysis of laboratory results and epidemiological survey results and environmental assessments, we judge this epidemic to be an outbreak of cutaneous anthrax, associated with slaughtering and other processes from infected cattle imported from other province. No cases have been reported in the area since 1995, this is the most reported outbreak involving the largest number of cases in recent years, analyze the cause of this year’s epidemic was the frequent movement of livestock across provinces increases the risk of importation. Plenty of rain this year, flooding occurred in some areas, which is conducive to the breeding of pathogens, higher temperature and humidity also favour the formation of anthrax spores, livestock may be at increased risk of contracting anthrax from soil due to increased exposure to spores in soil, dust through close grazing and looser soil [6], increased opportunities for pathogens to infect livestock.

Results of this investigation were consistent with other anthrax outbreak investigations in which patients were infected through contact with sick livestock or contaminated animal products [7–12]. Handling carcasses of livestocks with suspected and confirmed anthrax had been established as a risk factor for cutaneous anthrax [13, 14]. The outbreak occurred in July and August when many of the persons exposed to the sick cattle wore less clothing, the arms are completely exposed and ungloved, hence increasing potential skin contact and making exposure more likely. The majority of cases were adult males, consistent with the majority of adult males working in livestock slaughtering and livestock product processing. Vegetative forms of Bacillus anthracis are easily killed during normal cooking, but spores are much more resistant to adverse conditions and require heating to at least 100 °C for 15 min for inactivation [15]. Most of the livestock products have been recovered and destroyed, and those sold are cooked food, so there were no gastrointestinal anthrax cases identified.

As a result of our investigation, we have taken some corresponding measures, we do a good job in the treatment of the cases, Cutaneous anthrax is usually curable with prompt antibiotic therapy, approximately 10–20% of untreated patients die [16]. We also organize health monitoring and management of close contacts, close contacts are managed in home isolation, and a 3-day preventive medication (0.1 g levofloxacin, or 0.5 g ciprofloxacin) is implemented.

Disinfection experts formulated technical guidance and suggestions for home disinfection of Anthrax cases, organized on-site terminal disinfection and technical guidance, and carried out disinfection of home environment, breeding and slaughtering sites in H Village, P Village, and Z Village, where the cases occurred. The ground, walls, surfaces and air of the 13 places where the confirmed cases live should be sprayed with 1:5000 chlorine-containing disinfectant. The use of tableware and other daily necessities to implement immersion disinfection; use bleach powder to disinfect toilets, excreta, water and sewers; items such as clothes worn by confirmed patients before and after admission were burned. By August 24, 10,765 square meters of disinfection area had been completed.

Strengthening public health education. With a focus on the personnel engaged in breeding, slaughtering, processing, propaganda not contact, do not kill, do not eat, do not buy and sell, dead livestock and source of meat, raise awareness of the masses and from personnel of course of protective and prevent cause unnecessary panic, once found that appear the symptom of anthrax, immediately report to the local institution of disease prevention and control, and timely medical treatment.

We also made several recommendations to the local government, livestock anthrax which could be prevented by animal vaccination. Effective herd immunity against anthrax in cattle requires at least 80% of the animals in an area to be vaccinated [15]. During an outbreak, all livestock in areas around the affected area should be vaccinated, routinely vaccinate livestock from now on. Continue to strengthen public health education, the owners of livestock and butchers should also be educated regarding the need for personal protective equipment during the process of slaughtering and on health risks associated with slaughtering sick animals. Through the patient disease detection proved that animal quarantine loopholes need to enhance quarantine of livestock or their products transported from other places.

It is suggested to strictly regulate and crack down on the family slaughtering, and establish an effective supervision mechanism to prevent similar incidents happen again. Strengthen training and guidance for community-level medical personnel and raise the diagnosis level and awareness of zoonotic diseases, local medical institutions set up special expert teams to treat cases. An effective surveillance system should be established to facilitate early detection, control and prevention of anthrax outbreaks.

## Data Availability

The datasets generated during and analyzed during the current study are not publicly available due to it's a public health emergency but are available from the corresponding author on reasonable request.
